# RNA splicing patterns contribute to burst size variation among HIV-1-infected Jurkat cell clones

**DOI:** 10.1128/jvi.01334-25

**Published:** 2025-11-24

**Authors:** Kamya Gopal, Cleo Burnett, Siarhei Kharytonchyk, Ann Emery, Edmond Atindaana, Ronald Swanstrom, Jeffrey M. Kidd, Alice Telesnitsky

**Affiliations:** 1 Department of Microbiology & Immunology, University of Michigan Medical School 12266 , Ann Arbor, Michigan, USA; 2 Cellular and Molecular Biology Program, University of Michigan Medical School 12266 , Ann Arbor, Michigan, USA; 3 Lineberger Comprehensive Cancer Center, University of North Carolina at Chapel Hill 2331, Chapel Hill, North Carolina, USA; 4 Department of Biochemistry and Biophysics, University of North Carolina at Chapel Hill 318275, Chapel Hill, North Carolina, USA; 5 Department of Human Genetics, University of Michigan Medical School 21614, Ann Arbor, Michigan, USA; University Hospital Tübingen, Tübingen, Germany

**Keywords:** human immunodeficiency virus, RNA splicing, HIV-1 population dynamics, latency-reversing agents, HIV-1 expression parameters

## Abstract

Accurately quantifying virus release from HIV-1-infected cells is central to predicting infection outcomes and evaluating treatment strategies. Recent studies suggest that viral shedding can vary strikingly among infected cells. To identify predictors of virus release levels, a previously developed high-throughput molecular barcoding system was used to track the expression properties of individual infected clones within a polyclonal population. Consistent with previous reports, virus release spanned four orders of magnitude among clonal integrants. While reporter gene expression correlated poorly with virus release, intracellular viral RNA levels correlated well, and levels of unspliced HIV-1 RNA correlated most closely. Comparing clones with different virus release levels showed that they varied not only in total intracellular HIV-1 RNA levels but also in levels of viral RNA splicing. Remobilizing proviruses revealed that splicing differences were largely due to cell-intrinsic properties, although splicing differences were due to heritable features of the parent provirus for at least one clone. This clone displayed high levels of reporter gene expression from an HIV-1spliced RNA, but low levels of unspliced viral RNA and virus release. This over-splicing clone, which contained a non-synonymous substitution in *rev*, did not respond to treatment with the latency-reversing agent JQ1 but displayed increased virus release in response to treatment with pladienolide B, a splicing inhibitor. These findings demonstrate that splicing differences can contribute to virus production levels and suggest that future studies on proviral populations that include expanded clones may benefit from assessing clone-specific splicing properties.

**IMPORTANCE:**

Many models of HIV-1 infection rely on the assumption that actively infected cells release similar amounts of virus, despite recent reports that suggest shedding differs drastically among infected cells. In this study, the expression phenotypes of hundreds of integrant clones were analyzed to identify factors contributing to burst size variation. In agreement with previous reports, virus release spanned over four orders of magnitude within the infected pool. Both proviral expression levels and variation in splicing contributed to these burst size differences. While cell-intrinsic factors appeared to be the primary contributors to heterogeneous shedding patterns, viral point mutations were also observed and, in at least one case, contributed to particle release levels. Together, these findings demonstrate that expression variation among proviruses is both large and multifaceted and suggest that clone-specific differences in HIV-1 expression properties may contribute to unpredicted responses to treatment interventions.

## INTRODUCTION

HIV-1-infected cells are estimated to release on average between ~10^2^ and 10^4^ viral particles over their short lifetimes (1–4). Virus release levels can influence clinical outcomes associated with exponential HIV-1 outgrowth, such as HIV-1 expansion during acute infection (5), viral rebound after antiretroviral therapy (ART) interruption (6), and viral spread following reactivation from latency (7). Many current therapeutic strategies focus on perturbing the reservoir of latently infected cells that persist during ART (8–10), and the ability to accurately measure cell HIV-1 expression and virus release levels would be valuable in determining the efficacy of treatment interventions (11–15).

Recent efforts to characterize the expression profiles of individual patient-derived proviruses have revealed that both HIV-1 transcription levels and viral bursts are highly variable across integrants in cells of the same type (16–18). However, despite known burst size variability and recognition that persistent provirus populations are often dominated by a limited number of cell clones, persistent cell population sizes are often calculated by measuring viremia and assuming averaged virus release levels (19–21). Estimates of how many cells are contributing to virus release are central to predicting infection progression and evaluating therapeutic strategies and would be improved by a better understanding of proviral expression properties and their regulation.

A significant hurdle in estimating virus release among infected cells is that they sustain highly diverse levels of HIV-1 expression due to both host- and virus-mediated variables (22–24). Intracellular markers, such as HIV-1 RNA levels (12, 15, 25, 26), viral protein expression (27), and reporter gene expression (28–30), are typically used as measures for viral infection and replication, although consensus on an optimal predictor of virus release has yet to be achieved. Many virus expression models measure aggregate viral activity and fail to capture the inherent variability present across different proviruses, further complicating the identification of predictors of virus release.

Our lab has previously developed a high-throughput sequencing (HTS) system that enables tracking of HIV-1 integrants within a polyclonal pool of infected cells through molecular barcodes introduced into proviral genomes (31, 32). This experimental system is particularly well-suited to identifying correlates of virus release because multiple markers of infection, including viral RNAs and reporter gene expression, can be correlated to virus release for each of hundreds to thousands of individual proviruses within a polyclonal population. Additionally, individual integrant clones can be isolated from populations to identify mechanisms underlying provirus-specific differences in late replication steps.

In this study, HTS was used to investigate the spectrum of viral expression in a pool of Jurkat T cells infected with barcoded HIV-1 vectors. It was observed that virus release varied over 10,000-fold among integrant clones. Additional work showed that while multiple measures of reporter gene expression correlated poorly with virus release, intracellular levels of unspliced viral RNA were the strongest tested predictor of virus shedding.

## RESULTS

### Virus release levels span several orders of magnitude among integrant clones

**Fig 1:**
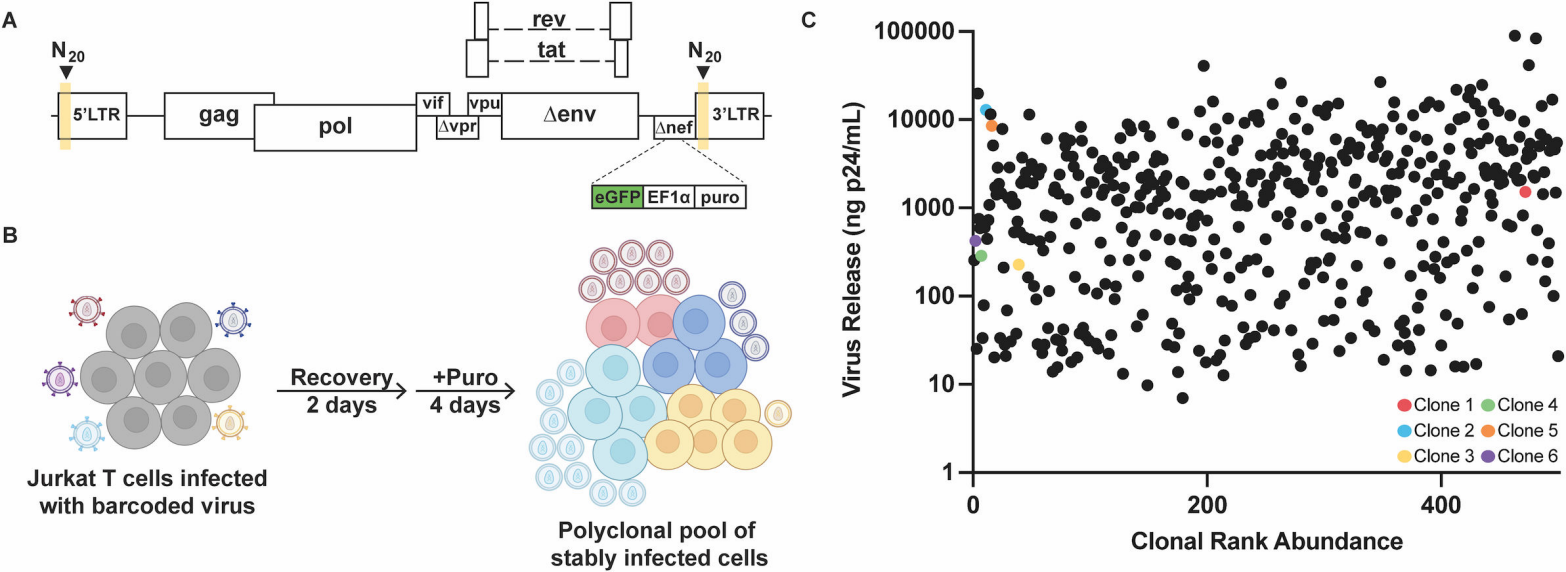
Virus release spans four orders of magnitude among integrants within a polyclonal pool of barcoded HIV-1 proviruses. (**A**) Schematic of this study’s HIV-1 vector. The yellow highlight depicts the position of the barcode present in the 3′UTR of the infecting virus and in both LTRs following reverse transcription. (**B**) Experimental workflow for generating a polyclonal pool of infected cells. (**C**) A scatterplot displaying virus release per cell as measured by HTS for the 500 most abundant clones in the polyclonal population. Clones are ranked on the x-axis by their abundance in the polyclonal pool, with the most abundant clone ranked 1. The six individual clones colorized in Panel **C** are described in Fig. 3.

To study differences in HIV-1 particle release, a pool of proviruses was established in the Jurkat T cell line using an HIV-1 NL4-3 strain-based library of *vpr*-, *env*-, and *nef*- vectors called HIV-GPV-, to reflect the presence of *gag* and *pol* but the absence of *vpr* (31, 32) (Fig. 1A). Each vector was engineered to contain a unique 20 base barcode in U3 to aid in tracking the expression properties of individual proviral lineages via HTS. Cells were infected at a multiplicity of infection less than 0.0001 to ensure that infected cells contained single proviruses (Fig. 1B). Provirus-containing cells were selected independently of HIV-1 LTR activity using a constitutive puromycin-resistance cassette. An enhanced green fluorescent protein (eGFP) gene in the *nef* open reading frame (ORF) facilitated the identification of cells in which the HIV-1 LTR promoter was transcriptionally active. Culturing in selection medium yielded a polyclonal pool of infected cells, which was expanded and then split into two parallel cultures that were analyzed separately to assess reproducibility.

First, variation in virus shedding was examined by determining the barcode content of extracellular viral particles and infected cell subpools. Barcodes were amplified from cDNA templated by virion RNA and from cell chromosomal DNA, then subjected to HTS. The fractional abundance of each barcode in the pool of extracellular viral particles released from the unsorted pool was first determined, and then virus release was normalized to clone size as determined by barcode abundance in cellular chromosomal DNA. Per cell virus release was plotted against clone size for the 500 most abundant infected cell clones (Fig. 1C). Consistent with previous reports (16, 32), virus shedding ranged over 4 orders of magnitude among the 500 analyzed clones (Fig. 1C). Later in this study, six individual clones were isolated for study from the infected pool via limiting dilution. The colorized data points shown in the graphs displaying population-level observations correspond to these isolated clones, which will be discussed below.

### Bimodal expression profiles and reporter gene expression levels correlate weakly with virus release

Having established that virus release levels differed 10,000-fold among proviruses in the infected pool, several measures of viral gene expression were then assessed to determine which best correlated with release levels. One expression parameter for at least some HIV-1 integrant clones is bimodal expression, which manifests as silent proviruses in a subset of daughter cells despite retention of LTR activity in sibling daughter cells (31–33). Indeed, phenotypic mixtures of both LTR-active and LTR-inactive cells have been observed within individual proviral clones both in infected patients and in experimental systems (31–33). Earlier studies on bimodal expression patterns revealed that the fractions of LTR-active and LTR-inactive cells in many integrant clones remain stable over at least 2 weeks (31).

**Fig 2:**
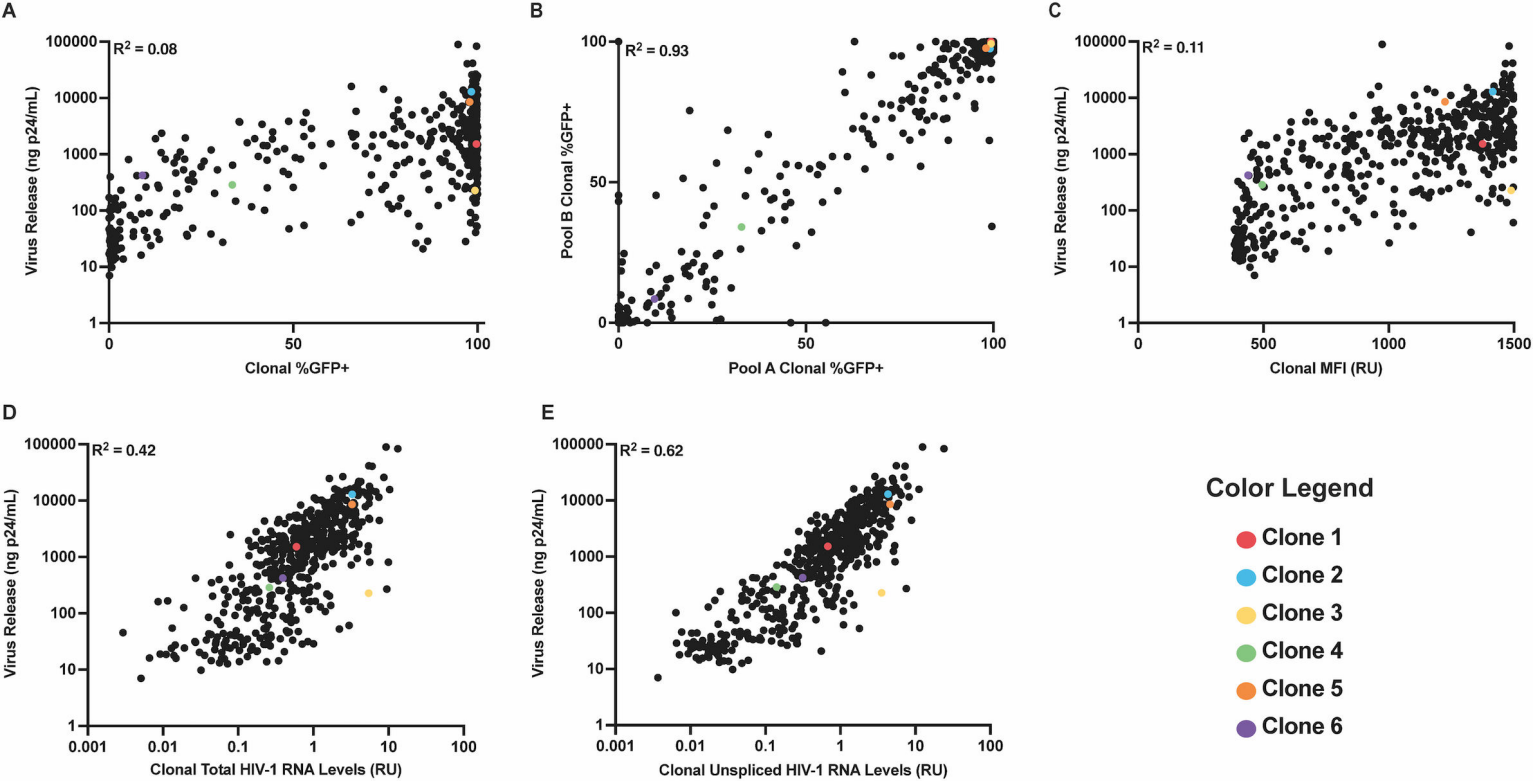
HIV-1 RNA levels correlate better with virus release than reporter gene expression. (**A**) A scatterplot comparing clonal bimodal expression patterns and virus release per cell for the 500 most abundant clones (r^2^ = 0.08, *P* < 0.0001). (**B**) A scatterplot comparing the bimodal expression patterns of the 500 most abundant clones between two replicate pools used in the present study (r^2^ = 0.93, *P* < 0.0001). (**C**) A scatterplot displaying the relationship between clonal average mean fluorescence intensity (MFI) values and virus release per cell for the 500 most abundant clones (r^2^ = 0.11, *P* < 0.0001). (**D**) A scatterplot displaying the relationship between intracellular HIV-1 RNA levels and virus release per cell for the 500 most abundant clones (r^2^ = 0.42, *P* < 0.0001). (**E**) A scatter plot comparing the relationship between unspliced HIV-1 RNA levels and virus release per cell for the 500 most abundant clones (r^2^ = 0.62, *P* < 0.0001). Note that the colorized clones are the same as those in Fig. 1 and will be further addressed in Fig. 3.

Here, the relationship between LTR-active proportion and virus release was examined. As noted above, the initial polyclonal pool of integrant clones was split into two prior to sorting, and these were cultured in parallel. Cells from each of these parallel cultures were sorted into eGFP^+^ (LTR-active) and eGFP^−^ (LTR-inactive) fractions using fluorescence-activated cell sorting. Barcodes were then amplified from the unsorted cells as well as from the eGFP^+^ and eGFP^−^ cell fractions. Bimodal expression was determined by calculating the fraction of cells with a given barcode sorted into the eGFP^+^ pool and normalizing to the total abundance of the barcode within both sorted pools. Although median burst size for <5% eGFP^+^ clones (28 ng p24/mL) was almost 100-fold less than that for >95% eGFP^+^ clones (2.4 µg p24/mL), when comparing bimodal expression patterns and per cell virus release, only a weak positive correlation (r^2^ = 0.08) was observed, suggesting that clonal LTR-active proportions were a poor predictor of burst size (Fig. 2A). Comparing bimodal expression patterns between the two subpools for the 500 most abundant clones revealed a strong correlation, thus demonstrating reproducibility (Fig. 2B).

**Fig 3:**
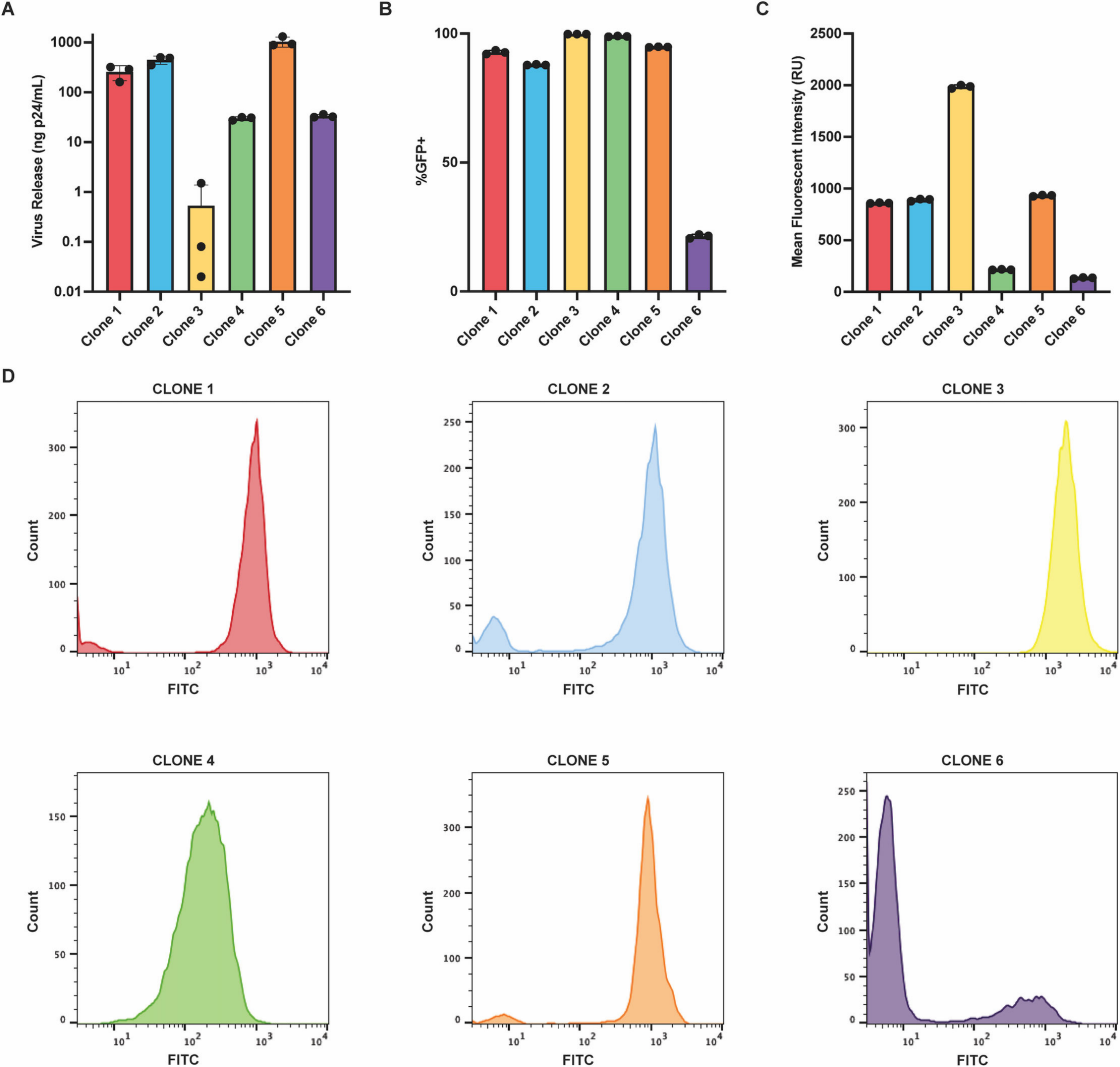
Virus release levels and reporter gene expression vary among isolated clones. (**A**) Virus release per 10^6^ cells, as measured by RT assay. (**B**) LTR-active proportions (%eGFP^+^) were determined for each clone using flow cytometry. (**C**) Clonal MFIs were measured by flow cytometry. (**D**) Representative histograms for each isolated clone depicting eGFP expression patterns.

Although bimodal expression was a poor predictor of clonal burst size, the possibility remained that eGFP expression levels might correlate more closely with virus release. It has been previously observed that LTR promoter activity can vary based on integration site (34), epigenetic modifications (35, 36), and transcriptional noise (37), among other factors. Therefore, a clone with a large fraction of LTR-active cells, each of which displayed low LTR activity, might express lower levels of eGFP than a clone with a smaller fraction of LTR-active cells, each displaying higher levels of LTR activity. To investigate the relationship between per cell eGFP expression levels and virus release, cells from the polyclonal pool were sorted based on mean fluorescence intensity (MFI) into low, intermediate, or high MFI subpools. To assign an approximate MFI value to each integrant clone, the fractions of its cells in the low, intermediate, and high MFI subpools were multiplied by the average MFI value for each subpool, as measured by flow cytometry. These calculations resulted in clonal MFI values, which are approximations and not absolute numbers. Plotting each clone’s calculated MFI against virus release per cell again revealed a weak positive correlation (r^2^ = 0.11) (Fig. 2C). Thus, per cell eGFP expression levels correlated more closely with virus release than bimodal expression patterns, but eGFP expression remained a poor predictor of virus release.

### Intracellular HIV-1 RNA levels, and particularly levels of unspliced RNA, correlate strongly with virus release

Next, the relationship between HIV-1 RNA levels and virus release was investigated. It was reasoned that measuring total intracellular viral RNA levels, unlike reporter gene expression, would account for all HIV-1 messages contributing to viral replication and might correlate more closely with virus release. To address this, barcode abundance in total cell RNA from the unsorted pools was normalized to barcode abundance in unsorted cell DNA. When plotting HIV-1 transcript levels per cell against virus release per cell for each clone, a stronger positive correlation emerged (r^2^ = 0.42), suggesting that HIV-1 RNA levels are a more accurate predictor of burst size than bimodal expression patterns or MFI (Fig. 2D).

Although total intracellular HIV-1 RNA correlated well with virus release, for a given amount of intracellular RNA (X-axis in Fig. 2D), virus release levels often ranged across two orders of magnitude (Y-axis in Fig. 2D), suggesting that additional parameters may contribute to burst size variation. One possible explanation was that RNA splicing product ratios were skewed. Transcription of integrated proviruses yields unspliced primary transcripts that can undergo alternative splicing and has been reported to generate more than 100 partially or completely spliced isoforms (38–40). It has been widely observed that unspliced HIV-1 RNA comprises the majority of viral transcripts in an infected cell, and that HIV-1 is more tolerant to a deficit of spliced transcripts than to unspliced transcripts (39). While maintaining both unspliced and spliced viral transcripts is essential for productive infection, unspliced RNA serves as mRNA for the structural Gag and Gag-Pol polyproteins and also as genomic RNA for progeny viral particles (39, 40). If splicing ratios were skewed, this might explain why reporter gene expression, which relied on one particular spliced RNA, did not map closely to virus release.

To address this, the relationship between unspliced HIV-1 RNA levels and virus release per cell was addressed. Unspliced viral RNA was selectively enriched from total cell RNA by affinity capture using biotinylated oligonucleotides complementary to a region of *gag* downstream of the 5′ major splice donor site, which is removed from all spliced HIV-1 RNAs (39, 41). Barcode abundance was determined by HTS, and the amount of each barcode in cell unspliced RNA was plotted against its prevalence in extracellular virion RNA (Fig. 2E). The results demonstrated that unspliced HIV-1 RNA levels were a superior predictor of virus release compared to total viral RNA levels (r^2^ = 0.62).

### Isolated clones also display expression variation

Individual integrant clones were then isolated via limiting dilution to validate population-level observations. Six clones were selected for further study: five of which exhibited similar high eGFP^+^ bimodal expression patterns but displayed dramatically different levels of virus release, and a sixth clone with fewer eGFP^+^ cells. The limited number of clones studied here was biased toward clones that displayed high %eGFP^+^ proportions because this allowed us to focus on clones with similar active cell populations but vastly different virus release levels. The lower %eGFP^+^ clone was included as a comparator. The colored dots in Fig. 2 designate the clones studied in isolation in Fig. 3. Virus release per 10^6^ cells (Fig. 3A), bimodal expression patterns (Fig. 3B), and eGFP expression (Fig. 3C and D) were characterized for these six isolated clones by quantifying RT activity and by flow cytometry. Some variations were observed between values determined for isolated clones and values for those same clones when assessed within the polyclonal population: notably, a twofold lower eGFP^+^ proportion for Clone 4 in the population context than when studied individually. Although the cause of this discrepancy was not fully characterized, it is noteworthy that Clone 4 LTR-active cells displayed very low eGFP levels (Fig. 3D), and thus some of the differences in bimodal proportion calculations for this clone may have reflected gating during population flow cytometry. However, for the most part, the expression trends determined for each clone were similar when studied within populations using HTS or when determined individually for the clones after isolation, further validating the approaches used in this study. Reflective of findings in the polyclonal population, the isolated clones had viral burst sizes that spanned approximately 3 orders of magnitude, with Clone 3 shedding the least virus and Clone 5 shedding the most (Fig. 3A). Notably, despite their large differences in virus release, Clones 1–5 displayed strikingly similar bimodal expression patterns, with >85% of daughter cells from each isolated clone exhibiting LTR activity (Fig. 3B).

The possibility that eGFP expression levels might correlate more closely with virus release was examined next using flow cytometry. Measuring eGFP expression by MFI revealed highly variable amounts of reporter gene expression among the clones (Fig. 3C and D). The MFI values from five of the six clones corresponded well with virus release levels, with one notable exception, Clone 3, which displayed very high MFI but very low virus release.

### Isolated clones displayed concordant differences in splicing levels and virus release

**Fig 4:**
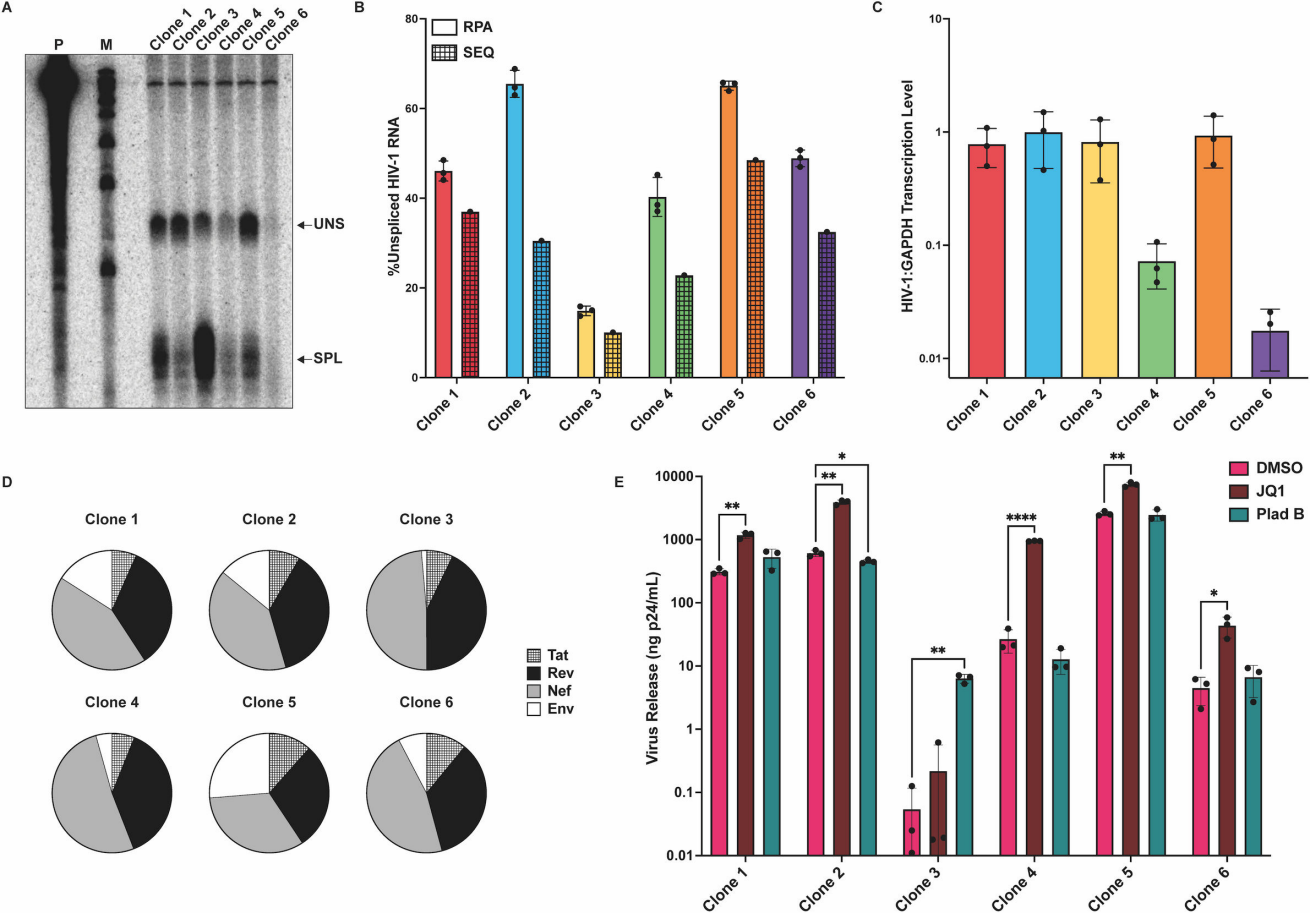
Clones isolated by limiting dilution from the polyclonal pool express different levels of unspliced and spliced viral transcripts and respond heterogeneously to LRA treatment. (**A**) Relative levels of HIV-1 unspliced (uns) and spliced (spl) RNA species were measured via RPA. (**B**) A bar graph comparing the relative production of unspliced HIV-1 RNA among clones. Data were compiled from quantification of RPA bands from experimental replicates of panel A (left bar for each clone) and data from HTS analysis (right bar for each clone). (**C**) Relative levels of HIV-1 RNA and cellular GAPDH RNA were measured via qPCR. (**D**) Pie charts depicting the relative abundance of spliced transcripts *tat*, *rev*, *nef*, and *env* for each clone as measured by a sequencing-based assay. (**E**) Isolated clones were treated with 0.1% DMSO, 2.5 µM JQ1, or 200 nM Plad B. Treatment effects on clonal virus release per 10^6^ cells were measured by RT assay. All statistical analyses were performed using Welch’s T test. * indicates *P* < 0.05, ** indicates *P* < 0.005, **** indicates *P* < 0.0001.

Next, relationships between cellular RNA levels and burst size were examined for the isolated clones. RNase protection assays (RPAs) were performed with total cellular RNA from each of the six clones (Fig. 4A and B) using a probe that spanned the major 5′ splice site and thus yielded distinct protected fragments for unspliced and spliced viral RNAs. Quantification of the levels of unspliced HIV-1 RNA per total viral transcripts (%unspliced HIV-1 RNA) revealed that Clone 3, the clone with the lowest virus release among those studied, had the lowest %unspliced HIV-1 RNA. Clones 2 and 5 had the highest %unspliced HIV-1 RNA and the highest virus release levels. The HIV-1 splice product trends revealed by RPA were consistent with those determined by an alternate approach to determining splicing levels, in which total cell RNA was subjected to an HTS-based assay (Fig. 4B and see Materials and Methods). Data from both RPA and HTS experiments were consistent with earlier reports that “over-splicing” negatively impacts virus production (42, 43).

Both Clone 4 and Clone 6 released relatively little virus despite having similar %unspliced HIV-1 RNA values to Clones 1, 3, and 5, which released several-fold more viral particles than Clones 4 and 6. To determine whether intracellular HIV-1 transcript levels could account for the discrepancy between %unspliced HIV-1 RNA and virus release, a quantitative PCR (qPCR) assay was performed. Relative RNA levels of GAPDH, a cellular housekeeping gene, and total HIV-1 RNA were determined using a probe set targeting GAPDH and the HIV-1 U5 region (Fig. 4C). The results indicated that Clones 4 and 6 expressed approximately 10- and 50-fold less HIV-1 RNA, respectively, than Clones 1, 3, and 5 when normalized to GAPDH, thereby reconciling the discordance between their virus release and %unspliced RNA levels. The relative amounts of specific splice classes were then determined. Using a previously described HTS-based assay on cell RNA isolated from the six clones, clone-specific differences were observed in amounts of intracellular RNAs containing splice junctions associated with *tat*, *rev*, *env*, and *nef* expression (Fig. 4D).

As demonstrated in this and earlier reports, over-splicing is deleterious to viral replication (42, 43). It remained unclear, however, whether suppressing over-splicing would reverse replication defects. To determine whether the over-splicing Clone 3 was otherwise competent to release virus, clones were treated with pladienolide B (44) (Plad B), which decreases splicing by inhibiting the U2 snRNP. In parallel, the clones were treated with the latency-reversing agent (LRA) JQ1 (45), a BET-family inhibitor (Fig. 4E). Virus release increased for most clones upon treatment with JQ1 but not Plad B, particularly for Clones 4 and 6, which expressed lower levels of HIV-1 RNA than all other clones. In contrast, Clone 3 did not respond to LRA treatment but exhibited an almost 250-fold increase in virus release following treatment with the splicing inhibitor Plad B.

### Cell-intrinsic differences predominate as determinants of interclonal variation in burst size and splicing, but heritable differences among proviruses also play a role

Having measured viral gene expression and particle release for isolated integrant clones, determinants of splicing differences among these integrants were then examined. Several factors known to contribute to differences in viral transcription, such as integration site, vary from one infected cell to another. It follows then that remobilizing virus from each integrant and infecting a fresh pool of cells would result in the averaging out of pronounced particle release and splicing phenotypes if these were characteristic of the integration sites of the parent proviruses or other cell-intrinsic differences, whereas differences would persist in progeny if phenotypes reflected viral mutations. Thus, to assess whether burst size determinants mapped to within proviral sequences or if cell-specific factors contributed to the distinct properties of infected clones, the integrated proviruses from each isolated clone were remobilized by transfecting integrant clones with a VSV-G expression plasmid, with Clone 3 additionally being treated with Plad B to increase virus release levels, and the properties of pooled progeny were studied.

**Fig 5:**
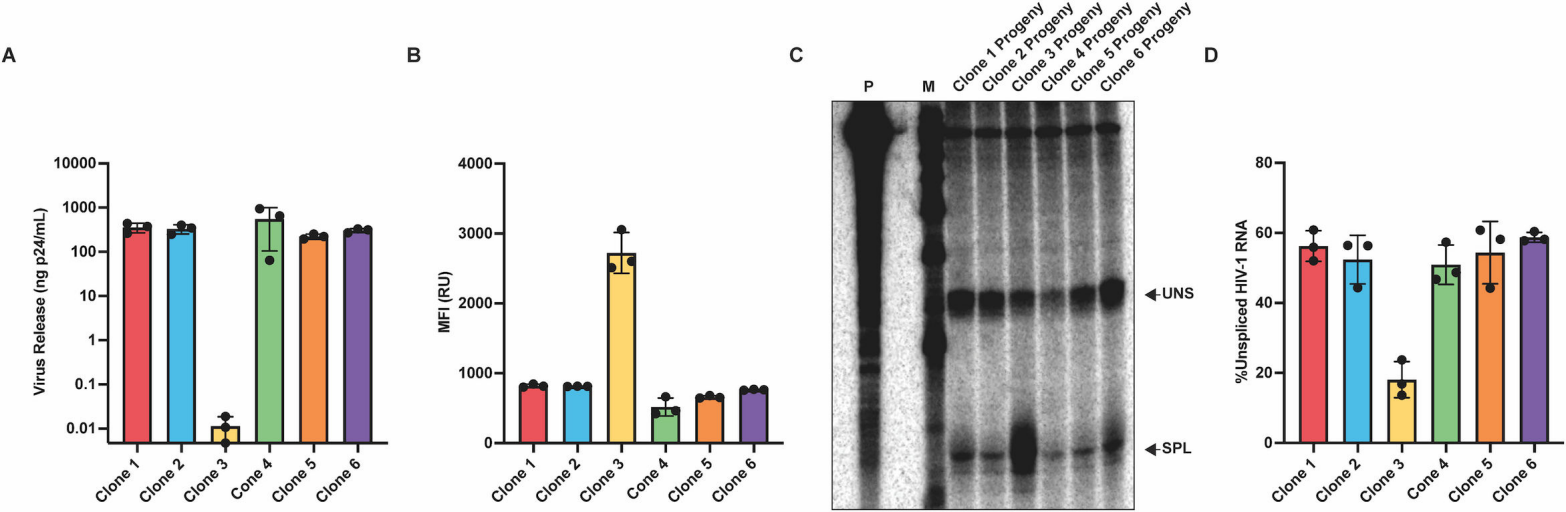
Cell-intrinsic and proviral sequence-specific determinants each contribute in part to differences in virus release among integrant clones. (**A**) Virus release per 10^6^ cells from populations infected with remobilized provirus from each isolated clone, as measured by RT assay. (**B**) MFIs of populations infected with remobilized proviruses were measured by flow cytometry. (**C**) Relative levels of HIV-1 unspliced (uns) and spliced (spl) RNA species were measured via RPA in populations infected with remobilized proviruses. (**D**) Quantification of RPA products from replicates of panel **C**.

After selection and expansion of polyclonal progeny provirus-containing cells, virus release was measured as previously (Fig. 5A). Interestingly, while virus release of the parent integrants spanned nearly three orders of magnitude (Fig. 3A), virus release of the newly infected cell pools differed by at most approximately 2-fold among progeny of Clones 1, 2, 4, 5, and 6. Cells infected with remobilized virus from Clone 3, however, had similarly low virus release levels compared to the parental clone.

Cells infected with remobilized virus were additionally subjected to eGFP expression analysis, which revealed changes in MFIs for all remobilization pools compared to the parent proviruses (Fig. 5B). Interestingly, MFI was higher in the remobilized Clone 3 pool compared to the MFIs of remobilized provirus from other pools. Additionally, when %unspliced RNA levels were determined for each remobilized clone, Clone 3 appeared to inherit the splicing patterns of its parent provirus and expressed approximately three times less unspliced HIV-1 RNA than the other remobilized clones (Fig. 5C and D).

Taken together, these data suggest that the variation in splicing and virus release observed above for Clones 4, 5, and 6 was not passed on through proviral sequences, while for Clones 1 and 2, the similarities in virus release between progeny pools and original clones do not clearly discriminate between hereditary and non-hereditary effects. On the other hand, splicing and virus release defects appeared to be heritable in the case of Clone 3, suggesting that proviral sequence differences may have contributed to its characteristic replication phenotypes.

### A rev mutation observed in the oversplicing *Clone 3* provirus is associated with reduced intracellular unspliced RNA levels

**Fig 6:**
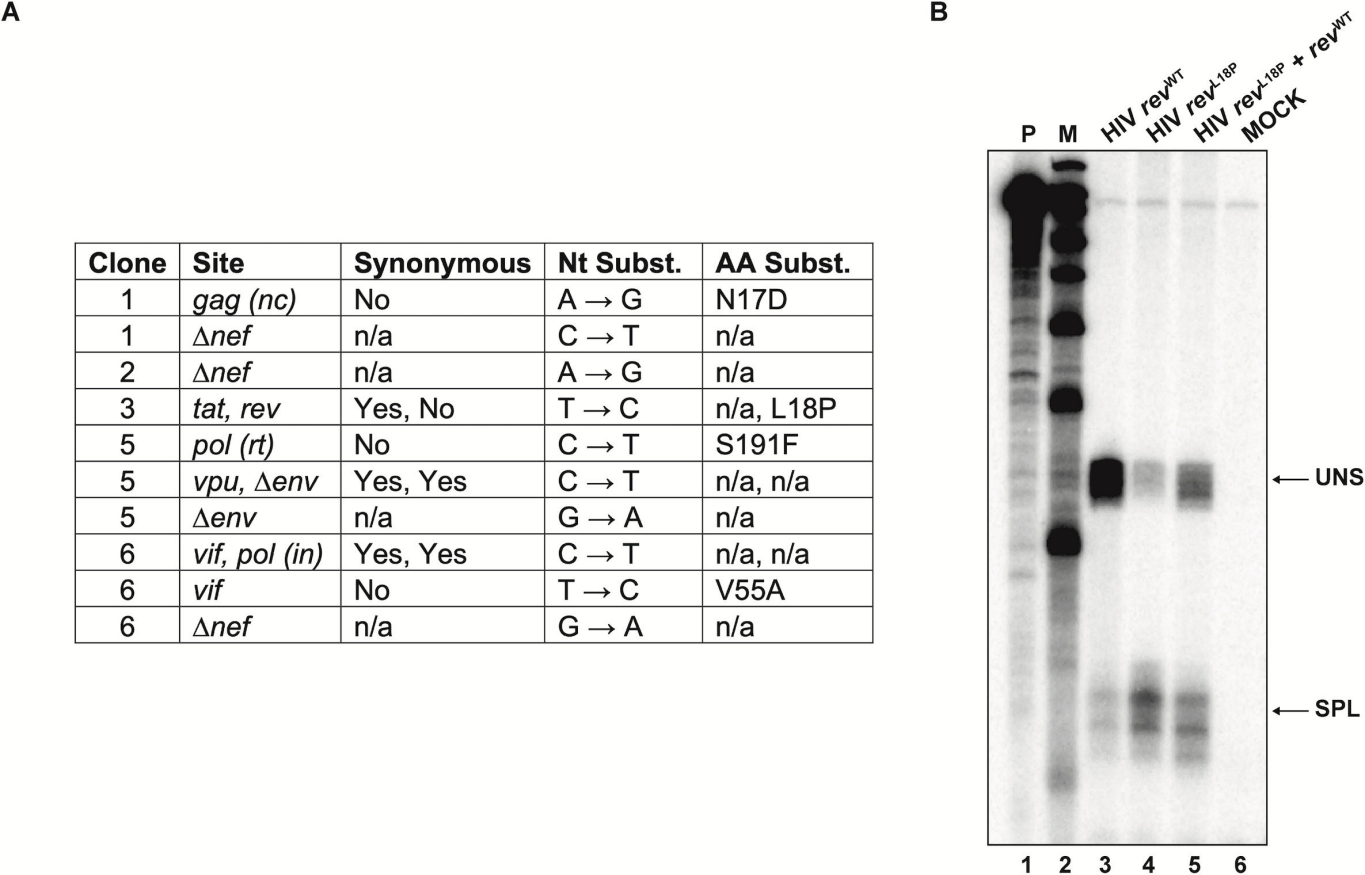
(**A**) Mutations identified in the proviruses of clones isolated via limiting dilution. (**B**) Relative levels of HIV-1 unspliced (uns) and spliced (spl) RNA species measured via RPA for cells transfected with the WT vector HIV-GPP (lane 3), HIV-GPP *rev*^L18P^ (lane 4), HIV-GPP *rev*^L18P^ co-expressed with pRev (lane 5), and in mock-transfected cells (lane 6).

Sequencing the proviruses in each of the parental clones revealed mutations had arisen in HIV-1 ORFs in all but one of the clones, presumably during the single cycle of reverse transcription involved in provirus genesis (Fig. 6A). The remobilization data above suggested that most of these mutations did not cause heritable changes in viral gene expression (Fig. 5A through D). However, because the progeny of Clone 3 retained the altered splice product ratios of the parent, any mutations in this provirus would be candidate modulators of splice product levels.

Sequencing the provirus in Clone 3 revealed that it contained a synonymous mutation in *tat* that was non-synonymous in the overlapping *rev* ORF, resulting in a leucine to proline substitution in Rev residue 18 (Fig. 6A). Leu-18 resides in an α helix that has previously been implicated in facilitating Rev multimerization (46). As such, a proline substitution could conceivably affect Rev function. It has been observed that defects in Rev can result in abnormal viral splicing product levels (47), which might explain the unusual splicing phenotype of Clone 3.

To test whether or not Clone 3 splice product defects could be attributed to the L18P substitution, intracellular spliced and unspliced product ratios for HIV-GPP, an NL4-3 strain-based *vpr*-, *env*-, and *nef*- vector with intact *rev*, were compared to those of HIV-GPP *rev*^L18P^, a modified version of HIV-GPP that contained the Clone 3 *rev* mutation, using an RPA (Fig. 6B). HIV-GPP is a vector similar to HIV-GPV- that does not express Vif or eGFP (48). HIV-GPP is not barcoded and was used to simplify procedures for this experiment, which did not examine expression at the level of individual proviruses. Whereas the RPA results on extracts of cells transfected with HIV-GPP confirmed the presence of high levels of unspliced RNA, similar to those observed for most of the remobilized vectors above, extracts from the cells transfected with HIV-GPP *rev*^L18P^ displayed the strong oversplicing phenotype characteristic of Clone 3. Co-transfecting cells with equimolar HIV-GPP *rev*^L18P^ and pRev, a vector expressing intact wild-type Rev under the CMV promoter, led to an intermediate phenotype. Together, these results indicate that the presence of the L18P *rev* mutation was sufficient to explain the heritable defect in splice product ratios observed for Clone 3.

## DISCUSSION

Here, an HTS analysis pipeline was used to investigate correlates of virus release levels in a Jurkat T cell-based HIV-1 infection model, initially using a polyclonal proviral population and subsequently validating results using individual integrant clones. While reporter gene expression correlated poorly with virus release, intracellular unspliced HIV-1 RNA levels correlated well. Consistent with the presence of outliers in the polyclonal population, isolation of individual integrant clones from the polyclonal population revealed that clone-specific low levels of viral shedding occurred despite high reporter gene expression for one isolate. This phenotype was determined to be due to over-splicing, wherein this clone retained LTR activity but produced insufficient levels of unspliced HIV-1 RNA to support robust virus production. Inhibiting splicing increased virus release in the over-splicing clone, while treatment with an LRA that did not directly inhibit splicing had no effect. Wide variation in virus release among integrants has been described in previous reports by our and other groups (16, 32). The present work demonstrated that proviruses can differ in the amounts of viral transcript classes produced and that these differences can contribute to heterogeneity in viral shedding among integrants.

Modest splicing differences were observed among several clones (Fig. 4B). However, provirus-specific splicing and virus release levels of the isolated clones were largely lost following proviral remobilization (Fig. 5D), suggesting that cell-specific differences, and not proviral mutations, primarily contributed to these expression patterns. In contrast, the expression properties of the over-splicing clone were heritable, and sequencing analysis suggested that a non-synonymous point mutation in *rev* caused this unique phenotype. Engineering the implicated *rev* mutation into a proviral clone and comparing its splice product phenotype to that of the parental clone confirmed that this single substitution in Rev was sufficient to generate the observed over-splicing phenotype.

Interestingly, the Rev residue that was altered in this mutant, Leu18, is only about 70% conserved among natural subtype B isolates (49). Previous work comparing multimerization and functioning of wild-type Rev to that of Rev mutants has shown that L18T and L18Q produce dimeric instead of higher-order complexes with cognate RNAs and also reduce Gag expression levels (50–52). Although less disruptive than the proline substitution examined here, naturally arising Leu18 substitutions clearly modulate Rev function and are common enough that it has been suggested that they may confer a selective advantage, perhaps by delaying the onset of AIDS (50).

Note, however, that the oversplicing phenotype of this *rev* mutant does not imply that Rev functions in splicing. Many years ago, a report claimed that the viral protein Rev suppresses HIV-1 transcript splicing by inhibiting spliceosome formation (53), which at that time appeared consistent with an earlier report that demonstrated that mutations in *rev* can result in increased levels of fully spliced viral RNAs (47). However, subsequent work on the functioning of Rev in unspliced RNA nuclear export suggested that the low levels of unspliced RNA observed in the absence of Rev reflect the degradation of RNA that is retained in the nucleus (54). Although some subsequent work has described possible interactions of Rev with the host splicing machinery, the prevailing model for the role of Rev in modulating RNA isoform ratios remains the promotion of unspliced HIV-1 RNA stability as a result of its nuclear export (55).

The work here does not resolve whether provirus sequence-independent differences in HIV-1 gene expression were due to integration site features or to genetic or epigenetic differences among cell clones. Jurkat T cells, an immortalized cancer line, were used in this study and in many others to investigate facets of HIV-1 infection (23, 56–58). Although Jurkat cells are a valuable model system, an important consideration is that HIV-1 may behave differently in primary cells and in people living with HIV-1 (PLWH). Notably, Jurkat cells are hypotetraploid (59, 60). Although the library used here was established in Jurkat cells that underwent only limited passage after their purchase from ATCC, these cells are highly unstable, and thus it is possible that the host genomes of integrant clones may differ from one another in functionally significant ways. However, results generated using infected cells isolated from PLWH suggest that these cells also support a wide range of unspliced and spliced viral RNA levels (17), which indicates that differential splicing of HIV-1 transcripts may be meaningful in a physiologically relevant context as well.

This report presents several observations that may be relevant to the interpretation of previous studies. Constructs in which reporter genes are expressed from spliced transcripts are commonly used as a read-out of HIV-1 expression (22, 30, 32, 61). We have demonstrated that in Jurkat cells, expression of a reporter gene from an HIV-1 spliced transcript and virus release levels correlate poorly for many integrants. Additionally, this work provided evidence that cell-intrinsic factors and mutations accrued over the course of even a single round of reverse transcription may contribute to widely observed differences in the response of infected cells to LRA treatment (62, 63).

The work here advances understanding of the complex effects of HIV-1 splicing and its modulation. As has been demonstrated for 5′ splice site-defective proviruses in certain PLWH with non-suppressible viremia (64), proviruses with some types of splicing abnormalities may not be cleared by immune effectors and may be a source of ongoing viremia despite defects in virus replication. In contrast to the reduced splicing observed in those mutants, the current study demonstrates that some proviruses can display an over-splicing phenotype and suggests that such proviruses may not respond to traditional LRA treatment. Various reports have demonstrated that inhibiting the production of spliced transcripts significantly decreases levels of extracellular virus and that targeting splicing factors may serve as a therapeutic approach for HIV-1 infection(65–67). Although several reports show that over-splicing of the viral genome is detrimental to viral replication (39), promoting over-splicing has been less widely studied as an HIV-1 treatment. Intriguingly, while Plad B has previously been used as a latency-promoting agent (67), it may serve as a stimulator of virus release in circumstances wherein a provirus undergoes over-splicing, as seen in this study.

In summary, work here tracked facets of HIV-1 replication via virus barcodes and confirmed earlier findings that virus release levels per cell can vary by integrant over several orders of magnitude. Predictably, some component of HIV-infected Jurkat T cell burst size differences appeared due to clone-specific proviral transcription levels, since the low intracellular transcript levels of some clones were boosted by a transcription-enhancing LRA, and transcription levels were restored in remobilized progeny provirus pools. However, some of the differences in virus release, both in a polyclonal infected pool and in isolated clones, were found to correlate with differences in intracellular unspliced HIV-1 transcript levels. For some clones, their splicing differences were not maintained when proviruses were remobilized, while for another—most notably, a *rev* point mutant—aberrant phenotypes were maintained upon virus passage. Thus, both cell-intrinsic factors and mutations in proviral genomes contributed to differential splicing among infected Jurkat cell clones. Although the current study describes proviruses only in transformed tissue culture cells, cells comprising the latent reservoir in PLWH are also diverse, and some component of their differences may include parameters that could affect the different classes of HIV-1 spliced RNAs. For example, there is some evidence for the presence of a virus with an attenuated Rev in a subset of long-surviving untreated PLWH (68). Speculatively, such proviruses or other splicing-defective subsets of the latent reservoir may contribute to continued inflammation by producing spliced transcript-encoded proteins, while not releasing virus or responding to LRA treatment.

## MATERIALS AND METHODS

### Cells and plasmids

Human embryonic kidney (HEK) 293T cells and Jurkat T cells were obtained from ATCC and grown at 37°C and 5% CO_2_. 293T cells were cultured in Dulbecco’s Modified Eagle Medium supplemented with 10% fetal bovine serum (FBS), 50 µg/mL gentamicin, and 0.33 µg/mL amphotericin B, whereas Jurkat cells were cultured in Roswell Park Memorial Institute (RPMI) 1640 Medium containing the same. HIV-GPP *rev*^L18P^ was generated for this study. This construct was generated by subcloning an amplicon derived from the chromosomal DNA of Clone 3 containing the *rev* substitution into HIV-GPP.

Published plasmids used in this study include the following: HIV-GPV^-^(31), a vector derived from HIV-GKO (69) with functional *gag-pol*, *tat*, *rev*, *vif,* and *vpu*, an eGFP gene in the *nef* ORF, and a puromycin-resistance gene under the EF1a promoter; HIV-GPP, a *vpr*-, *env*-, and *nef*- vector derived from HIV-1 NL4-3 (70); pRev, a *rev* expression plasmid (71); and pHEF-VSVG (72), which expresses vesicular stomatitis virus G protein.

### Generation of barcoded libraries and infected polyclonal pools, and transfection of 293T cells

Barcoded libraries were constructed as previously described (32). Briefly, HIV-GPV^−^ was digested with ClaI and MluI, and the resulting ~11.4 kb fragment was gel-purified. Barcoded inserts were generated via PCR amplification of the U3 region of HIV-GKO, the parent of HIV-GPV^−^, using the following primers: 5′-GACAAGATATCCTTGATCTGNNNNNNNNNNNNNNNNNNNNGCCATCGATGTGGATCTACCACACACAAGGC-3′ (forward) and 5′- CGGTGCCTGATTAATTAAACGCGTGCTCGAGACCTGGAAAAAC-3′ (reverse), and the resulting ~300 bp PCR product was gel purified. The vector library was generated via Gibson assembly of the HIV-GPV^−^ vector backbone and the PCR-generated insert pool at a 1:5 molar ratio. The resulting barcoded library was pseudotyped with pHEF-VSVG and packaged in 293T cells.

Jurkat cells were incubated with barcoded virus-containing media and 0.5 µg/mL polybrene at 37°C and 5% CO_2_ for 5 hours. The infection mixture was removed, and cells were plated in fresh media as previously described (32). At 48 hours post-infection, infected cells were selected using 0.5 µg/mL puromycin for 4 days, then allowed to expand in selection-free media, yielding the polyclonal population of integrants used in this study. Where indicated, single clones were isolated from the polyclonal pool via limiting dilution.

To infect cells with remobilized provirus from single integrant clones, cells from each clone were pseudotyped with pHEF-VSVG using the Neon Electroporation System, pelleted, and resuspended in fresh culture media. The over-splicing clone was cultured in media supplemented with 200 nM Plad B to stimulate higher virus release. At 24 hours after media replacement, cells were pelleted, and virus-containing supernatants were passed through 0.22 micron filters. Virus-containing media were then used to infect Jurkat T cells as described above.

For the experiments in Fig. 6B, 293T cells were transfected or co-transfected as previously described (41), with 5 µg HIV-GPP, 5 µg HIV-GPP *rev*^L18P^, or 5 µg of HIV-GPP *rev*^L18P^ and 5 µg pRev.

### Flow cytometry and cell sorting

Jurkat cells were suspended in phosphate-buffered saline supplemented with 1% FBS prior to all flow cytometry experiments. Sorting was performed through the University of Michigan flow cytometry core on the Bigfoot Spectral Cell Sorter using the fluorescein isothiocyanate (FITC) channel. To analyze bimodal expression within the infected polyclonal population, cells from the infected pool were sorted into eGFP^+^ and eGFP^−^ subpopulations. Similarly, to analyze average MFIs, cell populations were sorted into low MFI, intermediate MFI, and high MFI subpopulations. eGFP expression levels (%eGFP^+^ and MFI) were measured using the FITC channel on a BD LSR Fortessa. All flow cytometry data were analyzed using FlowJo, version 10.10.0.

### Nucleic acid isolation and preparation for sequencing

RNA was isolated from cells and viral particles using Trizol LS Reagent and Trizol Reagent, respectively. Genomic DNA was isolated from cells using the Dneasy Blood & Tissue Kit. All isolations were performed as per the manufacturer’s protocol. Unspliced HIV-1 RNA was isolated from total cellular RNA fractions using a biotinylated oligo complementary to the pol region of the viral genome (5′-TTGGCCTTGCCCCTGCTTCTGTATTTCTGC/3BIO/-3′) as previously described (41). To prepare samples for HTS, barcodes were PCR-amplified directly from cellular DNA samples. For RNA, cDNA was generated using the SuperScript First-Strand Synthesis System, then barcodes were PCR-amplified from the cDNA. All PCRs to amplify barcodes were conducted using Phusion polymerase and the following primers with Illumina partial adapters: 5′- ACACTCTTTCCCTACACGACGCTCTTCCGATCTGCCTGGCTAGAAGCACAAGA (forward) and 5′- GACTGGAGTTCAGACGTGTGCTCTTCCGATCTTGCCAATCAGGGAAGTAGCC (reverse). PCRs were purified using NEB Monarch PCR Purification Kits and submitted to Azenta for next-generation sequencing.

### HTS sequencing and analysis

Barcodes were analyzed using previously developed tools implemented in Python (31, 32). After barcode clustering, the 500 most abundant barcodes present in the DNA of both replicate infected pools were used for further analysis.

To calculate virus release for each clone, barcode abundance in virion RNA was divided by barcode abundance in cellular DNA. RT assays were performed on media samples isolated from the infected unsorted pool, and a standard curve was used to determine p24 concentration in the unsorted pool (73). Relative per cell virus release was multiplied by the p24 concentration of the unsorted pool to calculate a virus release value for clones in the infected pool.

To calculate bimodal expression patterns (%eGFP^+^) for each clone, the following formula was used: *E_i_* = {(*G_i_* × *P*) / [(*G_i_* × *P*) + (*W_i_* × *Q*)]} ×100, where *E_i_* is the %eGFP^+^ of the barcode *i*, *G_i_* is the abundance of barcode *i* in the eGFP^+^ sorted pool, *P* is the fraction of cells sorted into the eGFP^+^ pool, *W_i_* is the abundance of barcode *i* in the eGFP^−^ sorted pool, and *Q* is the fraction of cells sorted into the eGFP^−^ pool. To calculate clonal average MFIs, the following formula was used: *F_i_* = [(*L_i_* × *A* × *X*) + (*M_i_* × *B* × *Y*) + (*H_i_* × *C* × *Z*)] / [(*L_i_* × *A*) + (*M_i_* × *B*) + (*H_i_* × *C*)], where *F_i_* is the average MFI of barcode *i*, *L_i_* is abundance of barcode *i* in the low MFI sorted subpopulation, *A* is the fraction of cells sorted into the low MFI subpopulation, *X* is the average MFI of cells in the low MFI sorted subpopulation as determined by flow cytometry after sorting, *M_i_* is the abundance of barcode *i* in the intermediate MFI sorted subpopulation, *B* is the fraction of cells sorted into the intermediate MFI subpopulation, *Y* is the average MFI of cells in the intermediate MFI sorted subpopulation as determined by flow cytometry after sorting, *H_i_* is the abundance of barcode *i* in the high MFI sorted subpopulation, *C* is the fraction of cells sorted into the high MFI subpopulation, and *Z* is the average MFI of cells in the high MFI subpopulation as determined by flow cytometry after sorting. To calculate the per cell total HIV-1 RNA for each clone, a barcode’s abundance in total cell RNA is divided by its abundance in the cell DNA fraction of the unsorted pool. To calculate per cell unspliced HIV-1 RNA, a barcode’s abundance in unspliced HIV-1 RNA was divided by its abundance in cellular DNA.

For the six isolated clones, splicing levels and final splice acceptors were quantified using HTS as previously described (74). The Illumina MiSeq 300 was used to perform sequencing, and the bcl2fastq pipeline (v.2.20.0) was used for data pre-processing. Splice site usage was analyzed using tools developed by the Swanstrom lab.

### Quantification of virus release

Virions were quantified using a previously described qPCR-based RT assay (73). Virion production was determined by comparison to a standard curve generated using standards prepared in parallel with known p24 concentrations.

### RNase protection assay

RPAs were performed as previously described (75, 76) using a probe that protected a 128 base region in unspliced HIV-1 RNAs and a 72 base region in spliced HIV-1 RNAs, using an Amersham Typhoon scanner and quantified using ImageQuant TL analysis software, version 10.2.

### Quantification of HIV-1 RNA levels relative to GAPDH RNA

The relative amounts of HIV-1 and GAPDH RNA in cellular RNA were quantified using a qPCR-based assay. First, cDNA was generated using the SuperScript First-Strand Synthesis System for RT-PCR kit. cDNA and a primer-probe set targeting either HIV-1 or GAPDH were then used in a qPCR assay. The following primers and double-quenched probe were used to target HIV-1: 5′-ACTAGGGAACCCACTGCTTA (forward), 5′-CACAACAGACGGGCACA (reverse), and 5′-/56-FAM/AGCTTGCCT/ZEN/TGAGTGCTCAAAGTAGT/3IABkFQ-3′ (probe). The primers and double-quenched probe that targeted GAPDH were as follows: 5′-GGTGTGAACCATGAGAAGTATGA (forward), 5′-GAGTCCTTCCACGATACCAAAG (reverse), and 5′-/56-FAM/AGATCATCA/ZEN/GCAATGCCTCCTGCA/3IABkFQ-3′ (probe).

### Stimulation with JQ1 and pladienolide B

To measure the effects of JQ1 or pladienolide B treatment on virus production, cells from each clone were pelleted and then resuspended in fresh media containing 2.5 µM JQ1, 200 nM Plad B, or 0.1% DMSO as a control. 24 hours after treatment, aliquots of virus-containing media were filtered through a 0.22 micron filter, then used in RT assays to assess particle release in response to each treatment.

### Sequencing of integrated proviruses

Genomic DNA from each clone was isolated as described above, and proviral sequences were amplified using the following primers: 5′- TCTCTCGACGCAGGACTCGGCTTG (forward) and 5′-TGAGGGATCTCTAGTTACCAGAGTC (reverse). Amplicons from each clone were subcloned using the Zero Blunt TOPO PCR Cloning Kit for Sequencing, and the resulting plasmids were submitted to Azenta for sequencing using the Plasmid-EZ service. Barcodes were confirmed for each clone prior to performing further sequence analysis.

## Data Availability

All sequence data were deposited to the GEO under accession number GSE289347.
